# 1,4-Dibut­oxy-2,5-bis­{(*Z*)-2-[4-(9*H*-carbazol-9-yl)phen­yl]ethen­yl}benzene

**DOI:** 10.1107/S1600536812000414

**Published:** 2012-01-11

**Authors:** Wen-Wen Fei, Rui Li, Zhen-Yu Wang, Jie-Ying Wu

**Affiliations:** a Department of Chemistry, Anhui University, Hefei 230039, People’s Republic of China, and Key Laboratory of Functional Inorganic Materials Chemistry, Hefei 230039, People’s Republic of China

## Abstract

The title compound, C_54_H_48_N_2_O_2_, lies about an inversion centre. The carbazole ring system makes dihedral angles of 58.43 (7) and 88.96 (7)°, respectively, with the adjacent and central benzene rings. The dihedral angle between the two benzene rings is 52.01 (8)°. In the crystal, mol­ecules are linked by pairs of C—H⋯O inter­actions, forming a tape along the *a* axis. The methyl group is disordered over two sets of sites with occupancies of 0.63 (3) and 0.37 (3).

## Related literature

For the crystal structures of related carbazole derivatives, see: Liu *et al.* (2007 ▶); Piotr (2011 ▶); Paital *et al.* (2007 ▶); Zhang *et al.* (2010 ▶). For applications of carbazole derivatives, see: Ravindranath (2007 ▶); Sun *et al.* (2011 ▶); Zhao *et al.* (2008 ▶).
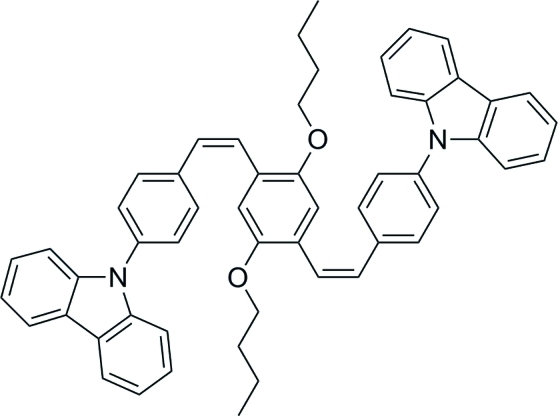


## Experimental

### 

#### Crystal data

C_54_H_48_N_2_O_2_
*M* *_r_* = 756.94
Monoclinic, 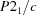

*a* = 8.437 (5) Å
*b* = 13.229 (5) Å
*c* = 19.165 (5) Å
β = 98.683 (5)°
*V* = 2114.5 (16) Å^3^
*Z* = 2
Mo *K*α radiation
μ = 0.07 mm^−1^
*T* = 293 K
0.30 × 0.20 × 0.20 mm

#### Data collection

Bruker SMART APEX diffractometer
Absorption correction: multi-scan (*SADABS*; Sheldrick, 1996 ▶) *T*_min_ = 0.979, *T*_max_ = 0.986
8735 measured reflections
3713 independent reflections
2969 reflections with *I* > 2σ(*I*)
*R*_int_ = 0.020

#### Refinement

*R*[*F* ^2^ > 2σ(*F* ^2^)] = 0.052
*wR*(*F* ^2^) = 0.153
*S* = 1.03
3713 reflections
274 parameters
32 restraints
H-atom parameters constrained
Δρ_max_ = 0.44 e Å^−3^
Δρ_min_ = −0.31 e Å^−3^

### 

Data collection: *SMART* (Bruker, 2007 ▶); cell refinement: *SAINT* (Bruker, 2007 ▶); data reduction: *SAINT*; program(s) used to solve structure: *SHELXS97* (Sheldrick, 2008 ▶); program(s) used to refine structure: *SHELXL97* (Sheldrick, 2008 ▶); molecular graphics: *SHELXTL* (Sheldrick, 2008 ▶); software used to prepare material for publication: *SHELXTL*.

## Supplementary Material

Crystal structure: contains datablock(s) I, global. DOI: 10.1107/S1600536812000414/is5035sup1.cif

Structure factors: contains datablock(s) I. DOI: 10.1107/S1600536812000414/is5035Isup2.hkl

Supplementary material file. DOI: 10.1107/S1600536812000414/is5035Isup3.cml

Additional supplementary materials:  crystallographic information; 3D view; checkCIF report

## Figures and Tables

**Table 1:** Hydrogen-bond geometry (Å, °)

*D*—H⋯*A*	*D*—H	H⋯*A*	*D*⋯*A*	*D*—H⋯*A*
C24—H24*B*⋯O1^i^	0.97	2.52	3.309 (4)	138

Symmetry code: (i) 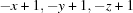.

**Fig. 1.**
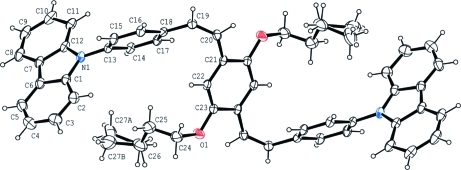
The molecular structure of the title molecule, showing 30% probability displacement ellipsoids.

## supplementary crystallographic information

### Comment

Recently, carbazole-based materials have been investigated for their high electrical and nonlinear optical properties (Ravindranath *et al.*, 2007; Sun *et al.*, 2011; Zhao *et al.*, 2008). The introduction about the structure of carbazole derivatives has been reported (Paital *et al.*, 2007; Piotr *et al.*, 2011). In the title molecule (Fig. 1), which is centrosymmetric, there are two 9-phenyl-9*H*-carbazole rings and a central benzene ring. In the crystal structure, the neighboring molecules are connected through weak intermolecular C—H···O interactions.

### Experimental

4-(9*H*-Carbazol-9-yl)benzaldehyde (0.30 g, 1.1 mmol), 2,5-dibutoxy-1,4-bis(triphenylphosphonium)benzene dichloride (0.42 g, 0. 5 mmol) and *tert*-BuOK (0.34 g, 3 mmol) were added to a mortar with grinding fully. The reaction residue was extracted with 200 ml of dichloromethane, washed four times with distilled water, and dried with anhydrous MgSO_4_. Then it was filtered and concentrated, purified by flash column-chromatography on silica. Elution with petroleum/ethyl acetate (50:1) gave the yellow powders (yield 69%).

### Refinement

All hydrogen atoms were placed in geometrically idealized positions and constrained to ride on their parent atoms, with C—H = 0.93–0.97 Å, and with *U*_iso_(H) = 1.2 or 1.5*U*_eq_(C). The bond lengths of C26—C27A and C26—C27B were restrained with C—C = 1.50 (2) Å. The anisotropic displacement parameters of atoms C25, C26, C27A and C27B were restrained by *DELU* and those of C25 and C26 were also restrained by *SIMU*.

### Crystal data

**Table:**

C_54_H_48_N_2_O_2_	*F*(000) = 804
*M**_r_* = 756.94	*D*_x_ = 1.189 Mg m^−^^3^
Monoclinic, *P*2_1_/*c*	Mo *K*α radiation, λ = 0.71069 Å
Hall symbol: -P 2ybc	Cell parameters from 3679 reflections
*a* = 8.437 (5) Å	θ = 2.4–27.0°
*b* = 13.229 (5) Å	µ = 0.07 mm^−^^1^
*c* = 19.165 (5) Å	*T* = 293 K
β = 98.683 (5)°	Needle, yellow
*V* = 2114.5 (16) Å^3^	0.30 × 0.20 × 0.20 mm
*Z* = 2	

### Data collection

**Table:**

Bruker SMART APEX diffractometer	3713 independent reflections
Radiation source: fine-focus sealed tube	2969 reflections with *I* > 2σ(*I*)
graphite	*R*_int_ = 0.020
φ and ω scans	θ_max_ = 25.0°, θ_min_ = 1.9°
Absorption correction: multi-scan (*SADABS*; Sheldrick, 1996)	*h* = −10→9
*T*_min_ = 0.979, *T*_max_ = 0.986	*k* = −15→15
8735 measured reflections	*l* = −22→18

### Refinement

**Table:**

Refinement on *F*^2^	Primary atom site location: structure-invariant direct methods
Least-squares matrix: full	Secondary atom site location: difference Fourier map
*R*[*F*^2^ > 2σ(*F*^2^)] = 0.052	Hydrogen site location: inferred from neighbouring sites
*wR*(*F*^2^) = 0.153	H-atom parameters constrained
*S* = 1.03	*w* = 1/[σ^2^(*F*_o_^2^) + (0.0789*P*)^2^ + 0.6687*P*] where *P* = (*F*_o_^2^ + 2*F*_c_^2^)/3
3713 reflections	(Δ/σ)_max_ < 0.001
274 parameters	Δρ_max_ = 0.44 e Å^−^^3^
32 restraints	Δρ_min_ = −0.31 e Å^−^^3^

### Special details

**Table:**

Geometry. All e.s.d.'s (except the e.s.d. in the dihedral angle between two l.s. planes) are estimated using the full covariance matrix. The cell e.s.d.'s are taken into account individually in the estimation of e.s.d.'s in distances, angles and torsion angles; correlations between e.s.d.'s in cell parameters are only used when they are defined by crystal symmetry. An approximate (isotropic) treatment of cell e.s.d.'s is used for estimating e.s.d.'s involving l.s. planes.
Refinement. Refinement of *F*^2^ against ALL reflections. The weighted *R*-factor *wR* and goodness of fit *S* are based on *F*^2^, conventional *R*-factors *R* are based on *F*, with *F* set to zero for negative *F*^2^. The threshold expression of *F*^2^ > σ(*F*^2^) is used only for calculating *R*-factors(gt) *etc*. and is not relevant to the choice of reflections for refinement. *R*-factors based on *F*^2^ are statistically about twice as large as those based on *F*, and *R*- factors based on ALL data will be even larger.

### Fractional atomic coordinates and isotropic or equivalent isotropic displacement parameters (Å^2^)

**Table:**

	*x*	*y*	*z*	*U*_iso_*/*U*_eq_	Occ. (<1)
C1	0.6314 (2)	0.01846 (14)	0.30709 (10)	0.0518 (5)	
C2	0.5261 (3)	0.09216 (18)	0.27681 (12)	0.0650 (6)	
H2	0.5617	0.1571	0.2689	0.078*	
C3	0.3666 (3)	0.0660 (2)	0.25877 (14)	0.0804 (7)	
H3	0.2937	0.1146	0.2390	0.096*	
C4	0.3127 (3)	−0.0308 (2)	0.26946 (15)	0.0855 (8)	
H4	0.2048	−0.0463	0.2562	0.103*	
C5	0.4158 (3)	−0.1037 (2)	0.29922 (13)	0.0761 (7)	
H5	0.3785	−0.1685	0.3063	0.091*	
C6	0.5790 (3)	−0.08014 (16)	0.31913 (10)	0.0569 (5)	
C7	0.7160 (3)	−0.13494 (14)	0.35341 (10)	0.0560 (5)	
C8	0.7409 (4)	−0.23444 (16)	0.37736 (12)	0.0703 (7)	
H8	0.6563	−0.2802	0.3724	0.084*	
C9	0.8896 (4)	−0.26362 (17)	0.40789 (12)	0.0774 (8)	
H9	0.9059	−0.3297	0.4239	0.093*	
C10	1.0173 (4)	−0.19693 (18)	0.41564 (11)	0.0735 (7)	
H10	1.1178	−0.2192	0.4367	0.088*	
C11	0.9989 (3)	−0.09695 (16)	0.39257 (10)	0.0599 (5)	
H11	1.0847	−0.0521	0.3976	0.072*	
C12	0.8465 (3)	−0.06768 (14)	0.36177 (9)	0.0504 (5)	
C13	0.8898 (2)	0.11459 (13)	0.33371 (9)	0.0459 (4)	
C14	0.9683 (2)	0.15220 (14)	0.39700 (9)	0.0487 (5)	
H14	0.9623	0.1178	0.4389	0.058*	
C15	0.9041 (2)	0.16441 (15)	0.27167 (10)	0.0529 (5)	
H15	0.8548	0.1384	0.2288	0.064*	
C16	0.9915 (2)	0.25288 (14)	0.27323 (9)	0.0510 (5)	
H16	1.0006	0.2857	0.2311	0.061*	
C17	1.0551 (2)	0.24032 (14)	0.39810 (9)	0.0477 (5)	
H17	1.1077	0.2646	0.4410	0.057*	
C18	1.0662 (2)	0.29390 (13)	0.33655 (9)	0.0426 (4)	
C19	1.1556 (2)	0.38949 (14)	0.33594 (9)	0.0490 (5)	
H19	1.2118	0.3978	0.2982	0.059*	
C20	1.1673 (2)	0.46543 (14)	0.38176 (10)	0.0497 (5)	
H20	1.2394	0.5161	0.3745	0.060*	
C21	1.0817 (2)	0.48059 (12)	0.44267 (9)	0.0432 (4)	
C22	0.9217 (2)	0.45503 (13)	0.43986 (10)	0.0464 (4)	
H22	0.8678	0.4248	0.3993	0.056*	
C23	0.8406 (2)	0.47336 (14)	0.49562 (10)	0.0492 (5)	
C24	0.5859 (3)	0.4184 (3)	0.43144 (16)	0.0973 (10)	
H24A	0.6265	0.4481	0.3914	0.117*	
H24B	0.4763	0.4411	0.4305	0.117*	
C25	0.5883 (4)	0.3084 (3)	0.42550 (18)	0.1024 (10)	
H25A	0.6980	0.2858	0.4264	0.123*	
H25B	0.5274	0.2883	0.3806	0.123*	
C26	0.5189 (4)	0.2581 (3)	0.48432 (18)	0.1114 (11)	
H26A	0.4070	0.2769	0.4817	0.134*	0.63 (3)
H26B	0.5754	0.2811	0.5294	0.134*	0.63 (3)
H26C	0.4259	0.2958	0.4938	0.134*	0.37 (3)
H26D	0.5976	0.2588	0.5268	0.134*	0.37 (3)
C27A	0.533 (2)	0.1370 (7)	0.4794 (7)	0.132 (5)	0.63 (3)
H27A	0.4529	0.1121	0.4426	0.198*	0.63 (3)
H27B	0.5179	0.1073	0.5236	0.198*	0.63 (3)
H27C	0.6376	0.1193	0.4690	0.198*	0.63 (3)
C27B	0.470 (3)	0.1500 (14)	0.4664 (14)	0.153 (8)	0.37 (3)
H27D	0.4231	0.1459	0.4176	0.230*	0.37 (3)
H27E	0.3938	0.1283	0.4956	0.230*	0.37 (3)
H27F	0.5632	0.1073	0.4745	0.230*	0.37 (3)
N1	0.7950 (2)	0.02509 (11)	0.33273 (8)	0.0504 (4)	
O1	0.68197 (18)	0.45206 (16)	0.49568 (9)	0.0871 (6)	

### Atomic displacement parameters (Å^2^)

**Table:**

	*U*^11^	*U*^22^	*U*^33^	*U*^12^	*U*^13^	*U*^23^
C1	0.0634 (12)	0.0469 (11)	0.0456 (10)	−0.0032 (9)	0.0100 (9)	−0.0028 (8)
C2	0.0711 (14)	0.0589 (13)	0.0640 (13)	0.0009 (11)	0.0070 (11)	0.0061 (10)
C3	0.0695 (15)	0.095 (2)	0.0752 (16)	0.0056 (14)	0.0056 (12)	0.0100 (14)
C4	0.0678 (15)	0.104 (2)	0.0837 (18)	−0.0165 (15)	0.0096 (13)	0.0063 (16)
C5	0.0846 (17)	0.0732 (16)	0.0732 (15)	−0.0259 (14)	0.0210 (13)	−0.0051 (13)
C6	0.0754 (14)	0.0500 (11)	0.0478 (10)	−0.0087 (10)	0.0177 (10)	−0.0043 (9)
C7	0.0882 (15)	0.0402 (10)	0.0438 (10)	−0.0057 (10)	0.0237 (10)	−0.0032 (8)
C8	0.116 (2)	0.0428 (12)	0.0564 (13)	−0.0053 (12)	0.0281 (13)	0.0017 (10)
C9	0.143 (3)	0.0413 (12)	0.0511 (12)	0.0125 (15)	0.0259 (14)	0.0067 (10)
C10	0.115 (2)	0.0589 (14)	0.0454 (11)	0.0290 (14)	0.0092 (12)	0.0029 (10)
C11	0.0848 (15)	0.0499 (12)	0.0441 (10)	0.0105 (11)	0.0071 (10)	−0.0027 (9)
C12	0.0774 (13)	0.0363 (10)	0.0385 (9)	0.0052 (9)	0.0122 (9)	−0.0012 (7)
C13	0.0582 (11)	0.0340 (9)	0.0455 (10)	0.0024 (8)	0.0084 (8)	−0.0016 (7)
C14	0.0693 (12)	0.0390 (10)	0.0381 (9)	0.0030 (9)	0.0095 (8)	0.0023 (7)
C15	0.0740 (13)	0.0453 (11)	0.0377 (9)	−0.0055 (9)	0.0029 (9)	−0.0057 (8)
C16	0.0727 (13)	0.0464 (11)	0.0350 (9)	−0.0033 (9)	0.0116 (9)	0.0009 (8)
C17	0.0627 (11)	0.0427 (10)	0.0361 (9)	0.0013 (9)	0.0026 (8)	−0.0040 (7)
C18	0.0502 (10)	0.0392 (9)	0.0398 (9)	0.0034 (8)	0.0111 (8)	−0.0030 (7)
C19	0.0568 (11)	0.0518 (11)	0.0397 (9)	−0.0054 (9)	0.0119 (8)	0.0003 (8)
C20	0.0573 (11)	0.0424 (10)	0.0497 (10)	−0.0099 (8)	0.0092 (9)	0.0016 (8)
C21	0.0541 (10)	0.0289 (9)	0.0456 (10)	0.0017 (7)	0.0039 (8)	0.0001 (7)
C22	0.0519 (10)	0.0389 (9)	0.0461 (10)	−0.0001 (8)	−0.0001 (8)	−0.0091 (8)
C23	0.0473 (10)	0.0431 (10)	0.0564 (11)	−0.0025 (8)	0.0051 (8)	−0.0098 (8)
C24	0.0584 (14)	0.132 (3)	0.100 (2)	−0.0136 (15)	0.0099 (13)	−0.0564 (19)
C25	0.0849 (18)	0.121 (3)	0.106 (2)	−0.0070 (17)	0.0279 (16)	−0.0361 (19)
C26	0.0852 (19)	0.157 (3)	0.099 (2)	−0.029 (2)	0.0365 (17)	−0.028 (2)
C27A	0.159 (10)	0.136 (7)	0.109 (6)	−0.076 (6)	0.044 (6)	−0.010 (5)
C27B	0.104 (11)	0.203 (15)	0.153 (13)	−0.004 (10)	0.019 (9)	−0.083 (11)
N1	0.0648 (10)	0.0354 (8)	0.0499 (9)	−0.0006 (7)	0.0048 (8)	0.0012 (7)
O1	0.0524 (9)	0.1291 (16)	0.0816 (11)	−0.0258 (9)	0.0161 (8)	−0.0559 (11)

### Geometric parameters (Å, °)

**Table:**

C1—C2	1.386 (3)	C17—C18	1.391 (2)
C1—N1	1.396 (3)	C17—H17	0.9300
C1—C6	1.408 (3)	C18—C19	1.473 (3)
C2—C3	1.383 (3)	C19—C20	1.328 (3)
C2—H2	0.9300	C19—H19	0.9300
C3—C4	1.384 (4)	C20—C21	1.476 (3)
C3—H3	0.9300	C20—H20	0.9300
C4—C5	1.365 (4)	C21—C22	1.385 (3)
C4—H4	0.9300	C21—C23^i^	1.402 (3)
C5—C6	1.407 (3)	C22—C23	1.374 (3)
C5—H5	0.9300	C22—H22	0.9300
C6—C7	1.437 (3)	C23—O1	1.368 (2)
C7—C8	1.399 (3)	C23—C21^i^	1.402 (3)
C7—C12	1.406 (3)	C24—O1	1.438 (3)
C8—C9	1.358 (4)	C24—C25	1.461 (5)
C8—H8	0.9300	C24—H24A	0.9700
C9—C10	1.383 (4)	C24—H24B	0.9700
C9—H9	0.9300	C25—C26	1.502 (4)
C10—C11	1.396 (3)	C25—H25A	0.9700
C10—H10	0.9300	C25—H25B	0.9700
C11—C12	1.387 (3)	C26—C27B	1.512 (16)
C11—H11	0.9300	C26—C27A	1.611 (11)
C12—N1	1.390 (2)	C26—H26A	0.9700
C13—C15	1.380 (3)	C26—H26B	0.9700
C13—C14	1.384 (3)	C26—H26C	0.9700
C13—N1	1.427 (2)	C26—H26D	0.9700
C14—C17	1.375 (3)	C27A—H27A	0.9600
C14—H14	0.9300	C27A—H27B	0.9600
C15—C16	1.381 (3)	C27A—H27C	0.9600
C15—H15	0.9300	C27B—H27D	0.9600
C16—C18	1.390 (3)	C27B—H27E	0.9600
C16—H16	0.9300	C27B—H27F	0.9600
			
C2—C1—N1	129.80 (18)	C17—C18—C19	122.95 (16)
C2—C1—C6	121.6 (2)	C20—C19—C18	129.11 (17)
N1—C1—C6	108.58 (17)	C20—C19—H19	115.4
C3—C2—C1	117.8 (2)	C18—C19—H19	115.4
C3—C2—H2	121.1	C19—C20—C21	129.00 (17)
C1—C2—H2	121.1	C19—C20—H20	115.5
C2—C3—C4	121.6 (3)	C21—C20—H20	115.5
C2—C3—H3	119.2	C22—C21—C23^i^	117.90 (17)
C4—C3—H3	119.2	C22—C21—C20	121.78 (16)
C5—C4—C3	120.8 (2)	C23^i^—C21—C20	120.25 (17)
C5—C4—H4	119.6	C23—C22—C21	121.55 (17)
C3—C4—H4	119.6	C23—C22—H22	119.2
C4—C5—C6	119.5 (2)	C21—C22—H22	119.2
C4—C5—H5	120.2	O1—C23—C22	124.48 (17)
C6—C5—H5	120.2	O1—C23—C21^i^	114.95 (17)
C5—C6—C1	118.6 (2)	C22—C23—C21^i^	120.55 (17)
C5—C6—C7	134.4 (2)	O1—C24—C25	111.3 (3)
C1—C6—C7	106.96 (18)	O1—C24—H24A	109.4
C8—C7—C12	118.8 (2)	C25—C24—H24A	109.4
C8—C7—C6	134.0 (2)	O1—C24—H24B	109.4
C12—C7—C6	107.15 (17)	C25—C24—H24B	109.4
C9—C8—C7	119.5 (2)	H24A—C24—H24B	108.0
C9—C8—H8	120.3	C24—C25—C26	111.8 (3)
C7—C8—H8	120.3	C24—C25—H25A	109.3
C8—C9—C10	121.3 (2)	C26—C25—H25A	109.3
C8—C9—H9	119.4	C24—C25—H25B	109.3
C10—C9—H9	119.4	C26—C25—H25B	109.3
C9—C10—C11	121.5 (2)	H25A—C25—H25B	107.9
C9—C10—H10	119.2	C25—C26—C27B	111.8 (10)
C11—C10—H10	119.2	C25—C26—C27A	110.8 (5)
C12—C11—C10	116.8 (2)	C25—C26—H26A	109.5
C12—C11—H11	121.6	C27A—C26—H26A	109.5
C10—C11—H11	121.6	C25—C26—H26B	109.5
C11—C12—N1	129.10 (19)	C27A—C26—H26B	109.5
C11—C12—C7	122.12 (19)	H26A—C26—H26B	108.1
N1—C12—C7	108.74 (18)	C25—C26—H26C	109.3
C15—C13—C14	119.39 (17)	C27B—C26—H26C	109.3
C15—C13—N1	120.42 (16)	C25—C26—H26D	109.3
C14—C13—N1	120.19 (16)	C27B—C26—H26D	109.2
C17—C14—C13	120.11 (17)	H26C—C26—H26D	107.9
C17—C14—H14	119.9	C26—C27A—H27A	109.5
C13—C14—H14	119.9	C26—C27A—H27B	109.5
C13—C15—C16	120.11 (17)	C26—C27A—H27C	109.5
C13—C15—H15	119.9	C26—C27B—H27D	109.5
C16—C15—H15	119.9	C26—C27B—H27E	109.5
C15—C16—C18	121.32 (17)	H27D—C27B—H27E	109.5
C15—C16—H16	119.3	C26—C27B—H27F	109.5
C18—C16—H16	119.3	H27D—C27B—H27F	109.5
C14—C17—C18	121.50 (16)	H27E—C27B—H27F	109.5
C14—C17—H17	119.2	C12—N1—C1	108.55 (16)
C18—C17—H17	119.2	C12—N1—C13	125.92 (17)
C16—C18—C17	117.49 (17)	C1—N1—C13	125.37 (15)
C16—C18—C19	119.55 (16)	C23—O1—C24	119.20 (18)
			
N1—C1—C2—C3	−177.0 (2)	C15—C16—C18—C17	2.6 (3)
C6—C1—C2—C3	0.2 (3)	C15—C16—C18—C19	−178.79 (18)
C1—C2—C3—C4	−0.9 (4)	C14—C17—C18—C16	−2.6 (3)
C2—C3—C4—C5	0.8 (4)	C14—C17—C18—C19	178.82 (17)
C3—C4—C5—C6	−0.1 (4)	C16—C18—C19—C20	143.1 (2)
C4—C5—C6—C1	−0.5 (3)	C17—C18—C19—C20	−38.4 (3)
C4—C5—C6—C7	177.0 (2)	C18—C19—C20—C21	−6.6 (3)
C2—C1—C6—C5	0.5 (3)	C19—C20—C21—C22	−38.6 (3)
N1—C1—C6—C5	178.24 (18)	C19—C20—C21—C23^i^	144.7 (2)
C2—C1—C6—C7	−177.64 (18)	C23^i^—C21—C22—C23	−0.4 (3)
N1—C1—C6—C7	0.1 (2)	C20—C21—C22—C23	−177.11 (17)
C5—C6—C7—C8	3.4 (4)	C21—C22—C23—O1	178.50 (19)
C1—C6—C7—C8	−178.8 (2)	C21—C22—C23—C21^i^	0.4 (3)
C5—C6—C7—C12	−177.1 (2)	O1—C24—C25—C26	62.0 (3)
C1—C6—C7—C12	0.6 (2)	C24—C25—C26—C27B	161.1 (12)
C12—C7—C8—C9	0.0 (3)	C24—C25—C26—C27A	−176.4 (8)
C6—C7—C8—C9	179.4 (2)	C11—C12—N1—C1	179.11 (18)
C7—C8—C9—C10	−0.2 (3)	C7—C12—N1—C1	1.2 (2)
C8—C9—C10—C11	0.1 (3)	C11—C12—N1—C13	−5.4 (3)
C9—C10—C11—C12	0.2 (3)	C7—C12—N1—C13	176.77 (16)
C10—C11—C12—N1	−178.08 (18)	C2—C1—N1—C12	176.7 (2)
C10—C11—C12—C7	−0.4 (3)	C6—C1—N1—C12	−0.8 (2)
C8—C7—C12—C11	0.4 (3)	C2—C1—N1—C13	1.1 (3)
C6—C7—C12—C11	−179.21 (17)	C6—C1—N1—C13	−176.38 (16)
C8—C7—C12—N1	178.43 (17)	C15—C13—N1—C12	125.6 (2)
C6—C7—C12—N1	−1.2 (2)	C14—C13—N1—C12	−54.4 (3)
C15—C13—C14—C17	2.2 (3)	C15—C13—N1—C1	−59.6 (3)
N1—C13—C14—C17	−177.80 (17)	C14—C13—N1—C1	120.4 (2)
C14—C13—C15—C16	−2.2 (3)	C22—C23—O1—C24	−7.1 (4)
N1—C13—C15—C16	177.77 (17)	C21^i^—C23—O1—C24	171.1 (2)
C13—C15—C16—C18	−0.2 (3)	C25—C24—O1—C23	90.2 (3)
C13—C14—C17—C18	0.3 (3)		

Symmetry codes: (i) −*x*+2, −*y*+1, −*z*+1.

### Hydrogen-bond geometry (Å, °)

**Table:**

*D*—H···*A*	*D*—H	H···*A*	*D*···*A*	*D*—H···*A*
C24—H24B···O1^ii^	0.97	2.52	3.309 (4)	138

Symmetry codes: (ii) −*x*+1, −*y*+1, −*z*+1.
